# An Internet-Based Cognitive Behavioral Intervention for Adolescents With Anxiety Disorders: a Study Protocol for a Randomized Controlled Trial

**DOI:** 10.1192/j.eurpsy.2023.1815

**Published:** 2023-07-19

**Authors:** H. Skaarnes, N. M. Sørensen, J. J. Lomholt, M. Thastum, K. Mathiasen

**Affiliations:** 1 Center for Digital Psychiatry, Odense; 2 Center for Psychological Treatment of Children and Adolescents, Aarhus University, Aarhus, Denmark

## Abstract

**Introduction:**

Anxiety disorders are the most prevalent mental health conditions among children and adolescents. However, it is estimated that less than 25% of all children and adolescents with an anxiety disorder receive professional help. Thus, it is of utmost importance to develop novel interventions that aim to increase treatment accessibility.

**Objectives:**

The aim of this study is threefold, to determine the effectiveness of CoolMinds, an iCBT intervention for adolescents with anxiety disorders. In addition to investigate predictive factors and the networks between symptoms, severity and change from pre- to post- treatment.

**Methods:**

The study is designed as a three-armed randomized controlled trial comparing iCBT with planned feedback, iCBT with on-demand help and a waitlist control, with 56 patients in each group. The participants in the two treatment conditions will receive 12 weeks of iCBT, while participants in the waitlist control wait for 12 weeks, before receiving iCBT with planned feedback. The participants in the two iCBT conditions will be randomized to get a booster session or not, 12 weeks after finishing treatment. The participants are adolescents between the age of 12 and 17 years and their parents. The families must live in the Region of Southern Denmark, and the adolescents must have a principal anxiety diagnosis according to DSM-5 criteria. The primary outcome measure are the Youth Online Diagnostic Assessment - child and parent versions. Outcomes will be evaluated at baseline, post-treatment and at the 3-, 6- and 12-month follow-ups. Symptoms of anxiety and depression are also measured between each session with PHQ-9 and S-SCAS.

**Results:**

The results from this study will be submitted to high-status international and peer-reviewed journals, as well as be presented at national and international conferences.

**Conclusions:**

This study will allow us to determine the efficacy of iCBT in adolescents with anxiety, where parent involvement is emphasized as part of the treatment. The results from this study intends to enhance accessibility of evidence-based treatment for adolescents with anxiety.

**Disclosure of Interest:**

None Declared

